# Annual acknowledgement of manuscript reviewers

**DOI:** 10.1186/1472-6890-14-2

**Published:** 2014-01-17

**Authors:** Magdalena Morawska

**Affiliations:** 1 BioMed Central, Floor 6, 236 Gray's Inn Road, London WC1X 8HB, UK

## Abstract

**Contributing reviewers:**

The editors of BMC Clinical Pathology would like to thank all of our reviewers who have contributed to the journal in Volume 13 (2013).

## 

Abbas Yazdanbod

Iran

Adam Lacy-Hulbert

USA

Ajay Matta

Canada

Aleodor Andea

USA

Alexander Link

Germany

Ana Morales

USA

Ana Elizabete Silva

Brazil

Andrew Beck

USA

Andrew Young

USA

Andrew Landstrom

USA

Andries Bakker

Netherlands

Anita Hjelmeland

USA

Anna Batistatou

Greece

Anna Merino

Spain

Antje Bornemann

Germany

Antoinette Perry

Ireland

Antonio Macchiarulo

Italy

Armin Piehler

Norway

Benjamin Rybicki

USA

Beom Kyung Kim

South Korea

Brad Zacharia

USA

Caj Haglund

Finland

Carlos Perez-Plasencia

Mexico

Carmen Jeronimo

Portugal

Carol Briggs

UK

Chang Cheng-Chi

Taiwan

Chien-Feng Li

Taiwan

Christopher Hall

USA

Christopher Neal

UK

Daniel Candotti

UK

Deniz Tural

Turkey

Dimitrios Krikelis

Greece

Donald Love

New Zealand

Doris Mayr

Germany

Eiji Mekata

Japan

Eliana Bignotti

Italy

Emanuela Monari

Italy

Emilia Hadziyannis

Greece

En Qiang Chen

China

Erik Thunnissen

Netherlands

Erika Santos

Brazil

Eugenio Maiorano

Italy

Fabien Reyal

France

Faisal Sanai

Saudi Arabia

Fang-Ming Chen

Taiwan

Fay Betsou

Luxembourg

Fekadu Abebe

Norway

Felicity EB May

UK

Francois X Claret

USA

Frederik Verburg

Germany

Fumio Nomura

Japan

Gary Tse

Hong Kong

Genichiro Ishii

Japan

Giampaolo Papi

Italy

Haodong Xu

USA

Harriette Kahn

Canada

Haruhiko Sugimura

Japan

Helenice Gobbi

Brazil

Hiroaki Ito

Japan

Hiroshi Inagaki

Japan

Hojoon Sohn

South Korea

Isidro Machado

Spain

Jacques Bonneterre

France

Jan PA Baak

Norway

Jason Sicklick

USA

Jaume Ordi

Spain

Jean-Paul Feugeas

France

Jena French

USA

Jen Der Lin

Taiwan

Joana Paredes

Portugal

Jochen Jarausch

Germany

Joel Parker

USA

John Kalbfleisch

USA

Jose Echevarria

Spain

Julian Crasta

India

Junko Aida

Japan

Karen Brondum-Nielsen

Denmark

Kathleen Kelly

USA

Kazuhiko Inoue

Japan

Keiichi Sakai

Japan

Keitaro Matsuo

Japan

Ken Young

USA

Kenneth Witwer

USA

Khaled Barakat

Canada

Koji Ueda

Japan

Leimin Sun

China

Lin Shen

China

Lisong Shen

China

Louisy Huang

France

Lourdes Mengual

Spain

Lynette Sholl

USA

Mallikarjun Patil

India

Maria Salinas

Spain

Margaret Currie

New Zealand

Maria Notarnicola

Italy

Maria Guembe

Spain

Maria Jastrzebska

Poland

Maria De Fatima Sonati

Brazil

Maria-Dolores Chiara

Spain

Mario Plebani

Italy

Mark Wade

Italy

Marta Pineda

Spain

Masaaki Tatsuka

Japan

Masakazu Yashiro

Japan

Massimo Barberis

Italy

Maurizio Bevilacqua

Italy

Melissa Snyder

USA

Michael Emmert-Buck

USA

Michael Tachezy

Germany

Michael Linnebacher

Germany

Michiyo Higashi

Japan

Miguel Quintanilla

Spain

Mike Cornes

UK

Mitsuru Ishizuka

Japan

Mu Kuan Chen

Taiwan

Mu Shui Dai

USA

Najmunnisa Nasreen

USA

Nan Haw Chow

Taiwan

Nidhi Singla

India

Norma Amador

Mexico

Paul Waring

Australia

Paul Cottu

France

Pengfei Shao

China

Peter Rambau

Tanzania

Pierre Saintigny

USA

Prashant Bavi

Saudi Arabia

Rafael Zaragoza

Spain

Renato Mariani-Costantini

Italy

Richard Slavin

USA

Richard Riedel

USA

Ridha Oueslati

Tunisia

Robert Schmidt

USA

Ronald Van Eijk

Netherlands

Roni Falk

USA

Sally Stephenson

Australia

Sanjeeva Kalva

USA

Sarah Wordsworth

UK

Satish Viswanath

USA

Shantibhusan Senapati

India

Sharon Akrish

Israel

Shigeyuki Murono

Japan

Shu Chun Lin

Taiwan

Shuishen Zhang

China

Songsak Petmitr

Thailand

Sonia Lain

Sweden

Sonja E Steigen

Norway

Stefan Ambs

USA

Steven Berberich

USA

Sumiya Ishigami

Japan

Sybille Mazurek

Germany

Takao Kamai

Japan

Takuya Moriya

Japan

Tamara Lah

Slovenia

Thomas Metz

USA

Thomas Wex

Germany

Thomas Hänscheid

Portugal

Tomoo Iwakuma

USA

Ulrich Ronellenfitsch

Germany

Uttara Chatterjee

India

Vickram Ramkumar

USA

Victor LJL Thijssen

Netherlands

Vincent Cryns

USA

Viviana Ritacco

Argentina

Waranun Buajeeb

Thailand

Wei Lien Wang

USA

William Laskin

USA

Woo Ho Kim

South Korea

Xiaochun Bai

China

Yanjing Gao

China

Yun Fan Liaw

Taiwan

Zhi Li

China

Zhili Liu

China

## Pre-publication history

The pre-publication history for this paper can be accessed here:

http://www.biomedcentral.com/1472-6890/14/2/prepub

